# On the Variability of Microbial Populations and Bacterial Metabolites within the Canine Stool. An in-Depth Analysis

**DOI:** 10.3390/ani11010225

**Published:** 2021-01-18

**Authors:** Carlo Pinna, Carla Giuditta Vecchiato, Costanza Delsante, Monica Grandi, Giacomo Biagi

**Affiliations:** Department of Veterinary Medical Sciences, University of Bologna, via Tolara di Sopra 50, 40064 Ozzano Emilia (BO), Italy; carla.vecchiato2@unibo.it (C.G.V.); costanza.delsante2@unibo.it (C.D.); monica.grandi8@unibo.it (M.G.); giacomo.biagi@unibo.it (G.B.)

**Keywords:** faecal microbiota, stool collection, DNA extraction, qPCR, bacterial metabolites, *Canis lupus familiaris*

## Abstract

**Simple Summary:**

The present study investigated for the first time the impact that different sampling points have on the abundance of microbial populations and metabolites within the canine stool. We found that inner stool subsamples resulted in higher concentrations of bacterial metabolites but not of microbial populations. These findings suggest that stool subsampling is unlikely to represent the canine microbiota and metabolome uniformly. We believe that complete homogenisation of the whole stool prior to analysis may improve the final outcome when investigating the canine gut microbiome.

**Abstract:**

Canine faecal microbial populations and metabolome are being increasingly studied to understand the interplay between host and gut microbiome. However, the distribution of bacterial taxa and microbial metabolites throughout the canine stool is understudied and currently no guidelines for the collection, storage and preparation of canine faecal samples have been proposed. Here, we assessed the effects that different sampling points have on the abundance of selected microbial populations and bacterial metabolites within the canine stool. Whole fresh faecal samples were obtained from five healthy adult dogs. Stool subsamples were collected from the surface to the inner part and from three equally sized areas (cranial, central, caudal) along the length axis of the stool log. All samples were finally homogenised and compared before and after homogenisation. Firmicutes, Bacteroidetes, *Clostridium* cluster I, *Lactobacillus* spp., *Bifidobacterium* spp. and *Enterococcus* spp. populations were analysed, as well as pH, ammonia and short-chain fatty acids (SCFA) concentrations. Compared to the surface of the stool, inner subsamples resulted in greater concentrations of SCFA and ammonia, and lower pH values. qPCR assay of microbial taxa did not show any differences between subsamples. Homogenisation of faeces does not affect the variability of microbial and metabolome data. Although the distribution patterns of bacterial populations and metabolites are still unclear, we found that stool subsampling yielded contradictory result and biases that can affect the final outcome when investigating the canine microbiome. Complete homogenisation of the whole stool is therefore recommended.

## 1. Introduction

The pet gut microbiota and the bacterial metabolome are gaining broad attention for their fundamental role in maintaining and promoting host health [1,2]. The alteration in the composition and richness of the intestinal microbiota is defined “dysbiosis” [3] and has been associated with several gastrointestinal disorders [4,5,6,7]. Due to ethical reasons and the feasibility of collection of the samples, characterization of the intestinal microbial community is most often conducted on faecal specimens by molecular tools, which requires the bacterial DNA extraction from the faecal matrix. Several authors reported discrepancies in the abundances of bacterial taxa between studies, attributed mainly to inter-individual differences [8,9], different DNA extraction protocols and sample storage conditions [10,11,12], and the application of different Next Generation Sequencing protocols [13,14]. Few papers focused the attention on the impact that different sampling methods have on the variability of the human [15,16,17,18,19] and canine [20] stool microbial community, while only Gratton et al. highlighted that spot sampling of faecal specimens resulted in a high degree of metabolic variation within human stool [21]. In veterinary medicine, most of the studies that are available perform DNA extraction using small aliquots of faeces with no homogenisation procedure of the sample. Since most of the commercial kits available are optimized to work with a limited amount of sample (usually up to 200 mg), stool subsampling without homogenisation appears may not be representative of the stool as a whole. To date, there is a lack of knowledge with regards to the distribution of bacterial populations and microbial metabolites throughout the same faecal specimen in the canine species. Here, we tested the impact that different sampling points have on the abundance of selected bacterial taxa and bacterial metabolites within the canine stool. We compared the amount of microbial populations, ammonia, short-chain fatty acids and pH values among subsamples collected along the stool log (longitudinal subsamples), from the surface and the inner part of the stool (depth subsamples) and compared homogenised and non-homogenised subsamples.

## 2. Materials and Methods

### 2.1. Samples Collection

Whole stool samples were collected from five healthy client-owned adult dogs (Table S1) with no medical history of antibiotic usage six months prior. For the purpose of this study, only faecal samples with a normal consistency score (2.0–3.0) were used. Dogs with diarrhoea were excluded from the study. Since no dog’s diets or habits had been modified, an ethics statement was not required for this study. All samples were provided by private dog owners who consented to our use for research purposes. The owners of each dog enrolled in the study signed an informed owner consent form. Animals defecated close to the laboratory on a sterile aluminium tray; a researcher observed each evacuation in order to identified the caudal part of the stool log (defined as the part of the stool that had left the bowel first, while the latter was named as the cranial part). Stools were put on ice after collection and transferred to the laboratory to be weighed and immediately processed for chemical analyses and genomic DNA extraction.

### 2.2. Stool Subsampling

Fresh faecal samples were equally divided into three parts: caudal, central, and cranial. From each part, four subsamples were taken from the surface of the stool, while other four subsamples were taken from the inner part of the stool. Outer specimens were cut with a sterile scalpel from the surface of the stool (max 2 mm deep): two aliquots (180–200 mg) were put into 2 mL bead tubes for DNA extraction, while the remaining two aliquots (~500 mg) were put into 15 mL centrifuge tubes for pH, ammonia, and short-chain fatty acids determination. For the inner specimens, the same sampling scheme was applied. Inner subsamples were collected from the centre of the stool using a fresh spatula, taking care to not transfer faecal material from the surface to the inner. Subsequently, the remaining part of the stool was put into a plastic bag and thoroughly homogenised by Stomacher Lab-Blender 400 (Seward, London, UK) for 10 min. Further aliquots were taken from the thoroughly homogenised faecal material and aliquoted in the same way as described above. In order to minimize variability among samples, each subsampling operation as well as chemical and microbiological analyses were performed by the same researcher, while a second laboratory assistant performed the DNA extraction.

### 2.3. Stool Preparation for Chemical Analysis and pH Determination

Immediately after subsampling, 500 mg faecal aliquots were diluted 1:10 *w/v* with deionized water. Tubes were shaken for 15 min on orbital shaker and centrifuged at 12,000× *g* for 10 min at 4 °C. A 2-mL aliquot of supernatant was collected and stored at −80 °C for chemical analysis. Faecal pH was determined immediately on the remaining supernatant using a laboratory pH meter (SevenMulti, Mettler Toledo, Greifensee, Switzerland; accuracy ±0.01 pH), standardised in buffer at pH 4, 7 and 9 prior to the start of each measurement session.

### 2.4. Chemical Analysis

Faecal ammonia was determined colorimetrically, according to Pinna et al. (2018), using an enzymatic colorimetric test (Urea/BUN—Color; BioSystems S.A., Barcelona, Spain) [22]. The SCFA were separated on a 2-m glass column (inner diameter, 3 mm) of 10% SP-1000 +1% H_3_PO_4_ on 100/120 Chromosorb W AW with nitrogen as the carrier. The chromatograph was a Fisons HRGC MEGA 2 series 8560 with a flame ionization detector. The temperatures of the injector and detector were 200 °C, and the oven temperature was 155 °C. 2-ethylbutyric acid (Sigma Aldrich, Taufkirchen, Germany) was used as the internal standard [23]. The concentrations of ammonia and SCFA were determined in duplicate.

### 2.5. Microbial Analysis 

DNA was extracted from fresh faecal samples immediately after subsampling using the Norgen Stool DNA Isolation Kit (Norgen Biotek Corp., Thorold, ON, Canada), according to the manufacturer’s instructions, including a bead beating step. DNA purity and concentration were measured using a DS-11 DeNovix spectrophotometer (DeNovix Inc., Wilmington, DE, USA). Purified DNA was diluted to 50 ng/µL and stored at −20 °C until used for qPCR amplification. Quantification of Firmicutes and Bacteroidetes [24], *Clostridium* cluster I [25], *Lactobacillus* spp. [26], *Bifidobacterium* spp. and *Enterococcus* spp. [27] were evaluated by qPCR using specific oligonucleotides (Table S2). SYBR-based qPCR assays were performed following the run protocol described previously [23,28]. Each sample was run in duplicate while standard curves were run in triplicate.

### 2.6. Statistical Analysis

Prior to statistical analysis, for each tested parameter, results obtained from the two aliquots collected from the same area were compared by one-way ANOVA. Since significant differences were not observed for any of the measured parameters, to increase the robustness of the statistical model it was decided to use the mean values obtained from each aliquot for statistical data analysis. For the depth and longitudinal area subsampling evaluation, data were analysed by the General Linear Model procedure. The model included inner/outer subsampling, longitudinal subsampling and their respective interaction as fixed effects and the dog as random effect. The homogenised group was set as the control group and treatments were compared using one-way ANOVA with Dunnett test as post-hoc test. Levene’s test was used to determine whether variance differed between groups. Significance level was set at *p* < 0.05. All statistical computations were carried out with Statistica 10.0 (Stat Soft Italia, Padua, Italy).

## 3. Results

### 3.1. Faecal Concentration of Bacterial Metabolites and pH Are Affected by Inner and Outer Subsampling but Not by Longitudinal Subsampling

**Figure 1:**
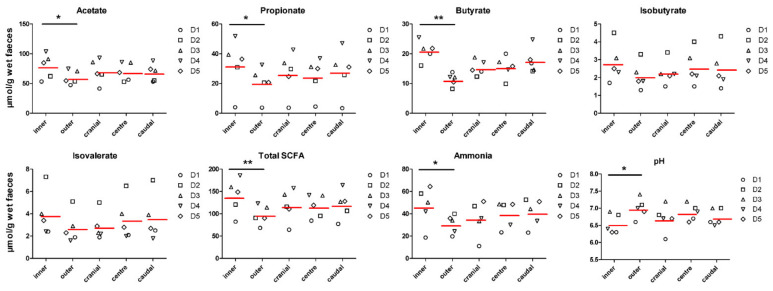
Short-chain fatty acids and ammonia concentrations, and pH values in stool subsamples collected from five different dogs. Concentrations of metabolites are expressed as µmol per gram of wet faeces. Red lines represent the mean of faecal SCFA and ammonia concentrations, and pH values. * *p* < 0.01 and ** *p* < 0.001. Dn = dog ID.

**Figure 2:**
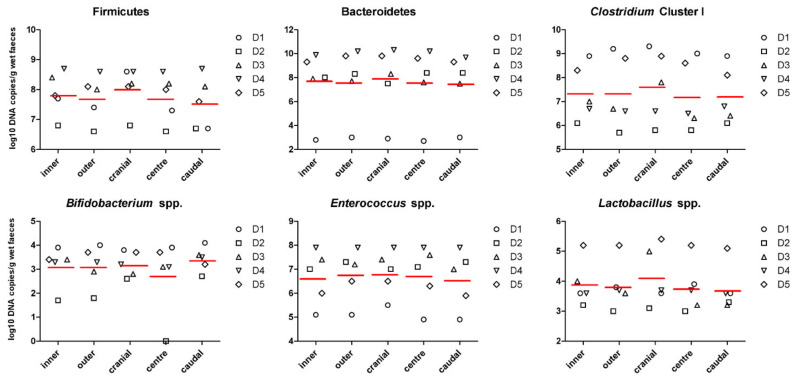
The abundances of selected bacterial taxa in stool subsamples collected from five different dogs. Values are expressed as log10 DNA copies per gram of wet faeces. Red lines represent the mean of log10 DNA. Dn = dog ID.

To assess whether the surface differs from the centre of the stool, specimens were collected from the inside and the outside the stool log. In addition, in order to determine longitudinal sampling variation, we compared subsamples collected along the stool log. Mean, standard deviation and *p*-values of inner and outer subsamples, and subsamples collected longitudinally are listed in Table 1. Significant differences were observed amongst metabolites across the stool log, but not along. Notably, mean concentrations of acetate (+31.2%; *p* = 0.002), propionate (+57.0%; *p* = 0.007), butyrate (+84.2%; *p* < 0.0001), total SCFA (+43.5%; *p* < 0.001), and ammonia (+51.6%; *p* = 0.003) were higher in the inner part of the stools compared to the outer part (Figure 1). Branched-chain fatty acids (BCFA) did not differ (*p* > 0.05) among groups, while pH was lower (6.54 vs. 6.99 for inner and outer subsamples, respectively; *p* = 0.001) in the inner part of the stools. The abundance of selected bacterial populations (Firmicutes, Bacteroidetes, *Clostridium* cluster I, *Bifidobacterium* spp., *Enterococcus* spp., *Lactobacillus* spp.) were not significantly different in inner and outer subsamples, neither in longitudinal subsamples (*p* > 0.05; Figure 2).

### 3.2. Homogenisation Has Minimal Effect on Metabolites Concentration

After the subsampling procedure, each stool was thoroughly homogenised and then subsampled twice for microbial and chemical analyses. Homogenised stool subsamples were arbitrarily set as the control and data were compared with those generated from specimens collected from each subsampling area (Table 2). Homogenised stools did not differ from non-homogenised subsamples collected along and across the stool log (*p* > 0.05), with the exception of caudal inner subsamples, which had higher concentrations of butyric acid (+42.2% compared to the homogenised stools; *p* < 0.0001). Valeric acid was only detected exclusively in the cranial inner part of dog D1 (data not shown). Results from Levene’s test showed that homogenisation technique does not affect the variability of bacterial metabolites, pH and microbial taxa when compared to non-homogenised subsamples (*p* > 0.05).

## 4. Discussion

Due to its huge variability and complexity, large-scale assessment of gut microbiota requires a large cohort of studies. Differences in data generated using different methodologies have been observed [16] and should be taken into account when extrapolating findings from different studies. Costea et al., focused the attention on the importance of DNA extraction on the outcome of molecular analysis and proposed a standardized DNA extraction method for human faecal samples to minimize the impact of technical variation in metagenomic analysis [11]. Defining a standardized methodology for the collection and preparation of faecal samples prior DNA extraction can be challenging, especially in the field of companion animals. When laboratory animals are not available, studies on gut microbiome are conducted using dogs owned by households, in which the owners are asked to collect and temporarily store the faecal sample in a domestic freezer until transport to the laboratory. Incomplete stool sample collection or late storage at freezing temperatures can occur; therefore, owner’s compliance represents another critical factor influencing results. While every effort was made to ensure reliability through technical replication, in this study we faced several limitations, first of all the limited number of subjects involved. Furthermore, the dogs were living in different environments and were fed a variety of diets, which may influence the interpretation of results.

In this study, we found large differences in number of metabolites between the inner and outer portion of stools, since inner subsamples resulted in greater concentrations of SCFA and ammonia compared to the surface of the stool. These findings were not observed previously by other studies. Although a small proportion of unabsorbed SCFA are excreted in the faeces [29], changes might be explained as a difference in absorption or utilization by the intestinal epithelial cells, as the external part of the stool is much more exposed to the gut mucosa. Another possible hypothesis, as pointed by Gorzelak et al. is that inner and outer portions represent distinct microenvironments harbouring different bacterial populations, leading to a non-homogeneous distribution of metabolites throughout the stool [15]. Moreover, we speculated that undigested nutrients are not homogeneously distributed throughout the stools, resulting in different microenvironments providing different energy sources for bacteria. Indeed, faeces are a complex matrix composed mainly of water, bacterial biomass and undigested food residue [30]. The diet is a factor that shapes the faecal output, faecal score and composition of faeces; with the increase of digestibility of the diet, faecal volume decreases significantly. In addition, a highly digestible pet food produces firm and well-formed faeces [31]. Non-degradable fibre intake is recognized to decrease digestibility and intestinal transit time and increase faecal volume and water content, increasing defecation frequency [32]. On the other hand, fermentable fibre has higher water-holding capacity, readily forms gels, increases luminal viscosity, and can be degraded by gut microbiota in the large bowel, resulting in significant concentrations of SCFA [33]. Thus, we speculated that different diets, and in particular the type and amount of fibre, can influence the spatial pattern of microorganisms and metabolites in the faeces. In this study, diets were not standardized among dogs. Other studies indicated that microbial metabolite oscillations are linked to host circadian rhythm and metabolism [34,35]. Therefore, multiple-timed and pooling vs. timed sampling appears to minimize variability originating from day to day variation in human samples [36,37]. To our knowledge there are no studies showing if canine faecal sample collection over time affects the faecal metabolic profile and microbiota compared to a single sampling.

In the present study, Firmicutes, Bacteroidetes, *Clostridium* cluster I, *Lactobacillus* spp., *Bifidobacterium* spp. and *Enterococcus* spp. populations did not differ among subsamples. Likewise, the differences of microbiota composition between the inner and outer layers of the human stool have been reported to be insignificant [19]. Due to the lower oxygen tension, we would expect an increase of anaerobic taxa in the inner part of the stools. In contrast to our findings, Gorzelak et al. found higher relative abundance of *Bifidobacterium* spp. and Firmicutes in the inner part of human stool [15]. In a study conducted with 30 female Beagles, Lin et al. found differences in the canine microbiota when comparing faecal aliquots [20]. However, none of the nine microbial genera who differed in the study by Lin et al. [20] were analysed in the present study, with the exception of Clostridia. The authors collected the fresh faecal specimens from both ends and the middle of each faecal sample, but they did not clarify if subsamples were collected from the centre or the surface of the stool. In our study, the composition of faecal microbial community was analysed by qPCR. Although the microbial groups selected for this study are representative of the main key taxa of the dog’s microbiota, it must be pointed that they do not fully represent the entirety of the microbiota. In contrast, Lin et al. observed changes in nine microbial genera using the Illumina MiSeq platform, which allowed a more comprehensive analysis of the complex and diverse faecal microbial communities [20].

In this study, homogenisation of faeces did not affect the microbial and metabolites variance within stool. Our results seem to contradict findings from other studies where relative abundances of bacterial taxa and metabolites concentrations were affected by homogenization [15,17,18,21]. Even though inter-individual variation in faecal microbiome among dogs appears to be greater than any differences associated with subsampling technique [8,9], since differences can occur when analysing samples collected from the inside rather than the outside of the stool, we can recommend that faecal samples used for bacterial metabolites assessment should be entirely collected and thoroughly homogenised. In studies investigating the gut microbiota and metabolome, dog faecal samples are usually collected by the owners and then stored frozen until processing. Since DNA extraction procedure usually requires the samples not to be thawed until the addition of lysis buffer, homogenisation procedure can be challenging or unsafe for the user (i.e., grinding in liquid nitrogen). In our study, fresh faecal specimens were homogenised by Stomacher; however, this procedure is, in many cases, not logistically feasible for researchers. When homogenisation step of the whole stool is not achievable, we recommend that the aliquot used for the metabolites determination consist of a cross-section of both the inner and outer part of the stool. The adoption of this simple protocol from the scientific community may improve the robustness of the canine faecal metabolome studies. 

## 5. Conclusions

This report is the first to provide information about the faecal distribution of bacterial taxa and metabolites in the canine species. Our results demonstrate that faecal subsampling affects the quantification of microbial metabolome throughout the stool. To increase the robustness of results, we recommend the homogenisation of the whole stool prior to analysis when investigating the gut microbiome.

## Figures and Tables

**Table 1:** Mean ±standard deviation (SD) values for short-chain fatty acids, ammonia, pH and microbial populations determined in depth and longitudinal stool subsamples collected from five different dogs.

	Depth Subsamples (*n* = 12)		Longitudinal Subsamples (*n* = 12)			ANOVA *p*-Value		
Item	Inner	Outer	Cranial	Centre	Caudal	Depth Sub.	Longitudinal Sub.	Depth × Longitudinal Sub.
acetate (µmol/g)	79.0 ± 20.0	60.2 ± 12.8	70.5 ± 20.9	69.8 ± 18.6	68.5 ± 19.6	0.002	0.954	0.725
propionate (µmol/g)	32.5 ± 16.8	20.7 ± 10.4	26.9 ± 15.1	24.8 ± 13.5	28.0 ± 17.6	0.007	0.809	0.834
butyrate (µmol/g)	21.0 ± 4.85	11.4 ± 3.03	15.3 ± 4.58	15.5 ± 6.13	17.7 ± 8.11	0.000	0.363	0.284
isobutyrate (µmol/g)	2.83 ± 1.11	2.09 ± 0.72	2.29 ± 0.74	2.57 ± 0.99	2.51 ± 1.27	0.058	0.815	0.930
isovalerate (µmol/g)	3.89 ± 2.08	2.74 ± 1.34	2.86 ± 1.31	3.50 ± 1.87	3.60 ± 2.25	0.099	0.627	0.916
total SCFA (µmol/g)	139.5 ± 37.9	97.2 ± 22.9	118.2 ± 38.9	116.3 ± 34.0	120.6 ± 43.2	0.000	0.932	0.646
ammonia (µmol/g)	46.7 ± 18.0	30.8 ± 8.81	35.7 ± 17.0	39.7 ± 14.2	40.9 ± 18.1	0.003	0.659	0.694
pH	6.54 ± 0.31	6.99 ± 0.32	6.69 ± 0.44	6.87 ± 0.33	6.73 ± 0.40	0.001	0.447	0.795
Firmicutes (log10 copies/g)	7.86 ± 0.85	7.74 ± 0.78	8.07 ± 0.72	7.74 ± 0.78	7.59 ± 0.90	0.667	0.353	0.907
Bacteroidetes (log10 copies/g)	7.57 ± 2.63	7.80 ± 2.72	7.77 ± 2.78	7.71 ± 2.80	7.58 ± 2.59	0.705	0.965	0.996
*Clostridium* cluster I (log10 copies/g)	7.40 ± 1.15	7.40 ± 1.49	7.67 ± 1.42	7.25 ± 1.39	7.27 ± 1.20	0.995	0.764	0.970
*Bifidobacterium* spp. (log10 copies/g)	3.14 ± 1.03	3.14 ± 1.00	3.22 ± 0.54	2.76 ± 1.50	3.42 ± 0.66	1.000	0.385	0.896
*Enterococcus* spp. (log10 copies/g)	6.68 ± 1.09	6.80 ± 1.03	6.86 ± 0.87	6.77 ± 1.15	6.60 ± 1.19	0.752	0.865	0.965
*Lactobacillus* spp. (log10 copies/g)	3.94 ± 0.82	3.86 ± 0.96	4.15 ± 0.95	3.80 ± 0.84	3.75 ± 0.88	0.789	0.448	0.677

**Table 2:** Mean, standard deviation (SD) and variance values for short-chain fatty acids, ammonia, pH and microbial populations determined in each subsampling area and in the homogenised stool collected from five different dogs.

Item		Homogenised	Cranial Inner	Cranial Outer	Centre Inner	Centre Outer	Caudal Inner	Caudal Outer	ANOVA *p*-Value	Levene’s *p*-Value
acetate (µmol/g)	mean ± SD	75.2 ± 17.0	77.2 ± 23.6	63.7 ± 17.8	79.3 ± 21.0	60.4 ± 10.9	80.5 ± 20.0	56.5 ± 10.1	0.200	
	variance	288.6	555.4	316.9	440.3	119.2	401.0	101.0		0.596
propionate (µmol/g)	mean ± SD	27.1 ± 14.1	32.0 ± 16.9	21.9 ± 12.7	29.9 ± 16.3	19.8 ± 8.96	35.6 ± 20.4	20.4 ± 11.5	0.531	
	variance	200.1	284.1	161.6	265.2	80.2	415.0	133.1		0.701
butyrate (µmol/g)	mean ± SD	16.9 ± 3.30 a	18.8 ± 3.16 a	11.8 ± 2.52 a	20.1 ± 4.72 a	11.0 ± 3.24 a	24.1 ± 5.63 b	11.3 ± 3.83 a	<0.0001	
	variance	10.9	10.0	6.37	22.3	10.5	31.7	14.7		0.074
isobutyrate (µmol/g)	mean ± SD	2.48 ± 0.87	2.60 ± 0.55	1.98 ± 0.83	2.90 ± 1.12	2.24 ± 0.81	2.98 ± 1.63	2.04 ± 0.65	0.567	
	variance	0.76	0.30	0.69	1.26	0.65	2.65	0.42		0.755
isovalerate (µmol/g)	mean ± SD	3.38 ± 1.70	3.31 ± 1.20	2.41 ± 1.38	4.00 ± 2.12	3.00 ± 1.67	4.37 ± 2.92	2.82 ± 1.17	0.664	
	variance	2.88	1.43	1.90	4.48	2.78	8.52	1.38		0.734
total SCFA (µmol/g)	mean ± SD	125.0 ± 29.8	134.6 ± 41.1	101.8 ± 32.3	136.2 ± 36.4	96.4 ± 16.9	147.8 ± 43.6	93.5 ± 21.4	0.073	
	variance	886.5	1686.0	1045.4	1323.4	285.5	1898.3	458.0		0.399
ammonia (µmol/g)	mean ± SD	40.2 ± 13.2	41.9 ± 20.3	29.4 ± 11.9	46.4 ± 15.7	32.9 ± 9.70	51.8 ± 20.2	30.0 ± 5.19	0.158	
	variance	175.0	412.1	142.1	245.2	94.0	409.8	26.9		0.721
pH	mean ± SD	6.79 ± 0.25	6.50 ± 0.39	6.87 ± 0.45	6.66 ± 0.31	7.08 ± 0.19	6.45 ± 0.25	7.02 ± 0.32	0.025	
	variance	0.06	0.15	0.20	0.10	0.04	0.06	0.10		0.284
Firmicutes (log10 copies/g)	mean ± SD	7.84 ± 0.71	8.14 ± 0.80	7.99 ± 0.72	7.87 ± 0.78	7.62 ± 0.85	7.57 ± 1.03	7.61 ± 0.87	0.905	
	variance	0.50	0.63	0.53	0.61	0.72	1.05	0.76		0.286
Bacteroidetes (log10 copies/g)	mean ± SD	7.67 ± 2.93	7.65 ± 2.96	7.89 ± 2.94	7.57 ± 2.86	7.85 ± 3.08	7.50 ± 2.70	7.66 ± 2.80	1.000	
	variance	8.59	8.77	8.64	8.17	9.47	7.30	7.83		0.957
*Clostridium* cluster I (log10 copies/g)	mean ± SD	7.52 ± 1.17	7.66 ± 1.25	7.69 ± 1.71	7.19 ± 1.42	7.32 ± 1.52	7.35 ± 0.93	7.18 ± 1.54	0.994	
	variance	1.37	1.57	2.94	2.03	2.31	0.86	2.38		0.388
*Bifidobacterium* spp. (log10 copies/g)	mean ± SD	3.27 ±0.47	3.12 ± 0.50	3.32 ± 0.61	2.75 ± 1.61	2.78 ± 1.58	3.54 ± 0.70	3.30 ± 0.66	0.843	
	variance	0.22	0.25	0.37	2.59	2.48	0.49	0.44		0.301
*Enterococcus* spp. (log10 copies/g)	mean ± SD	6.92 ±1.14	6.83 ± 0.81	6.88 ± 1.02	6.75 ± 1.21	6.79 ± 1.23	6.47 ± 1.40	6.74 ± 1.08	0.997	
	variance	1.30	0.66	1.05	1.47	1.52	1.97	1.17		0.895
*Lactobacillus* spp. (log10 copies/g)	mean ± SD	4.16 ±0.87	4.11 ± 0.94	4.20 ± 1.06	3.74 ± 1.00	3.85 ± 0.76	3.97 ± 0.62	3.54 ± 1.12	0.907	
	variance	0.75	0.89	1.12	1.00	0.58	0.39	1.25		0.948

## Data Availability

Not applicable.

## Supplementary Materials

The following are available online at https://www.mdpi.com/2076-2615/11/1/225/s1, Table S1: Signalment of the enrolled dogs; Table S2: Primers used in the qPCR assay.
