# Supplementary material for: On the Variability of Microbial Populations and Bacterial Metabolites within the Canine Stool. An in-Depth Analysis
Source: Animals (Basel). 2021 Jan 18;11(1):225. doi: 10.3390/ani11010225 (PMC7831317; doi:10.3390/ani11010225)
Supplement: Supplementary file 1 [file animals-11-00225-s001.pdf]

Table S1. Signalment of the enrolled dogs.

Dog	Breed	Sex	Age (yrs)	BW (kg)	Faecal mass (g)
D1 Cesare	French Bulldog	M	9	14.8	45.4
D2 Mandy	Pug	F(n)	9	8.0	40.0
D3 Tabata	Mixed	F(n)	6	28.5	98.4
D4 Nina	Mixed	F(n)	7	23.0	110.5
D5 Brenno	Boxer	M	6	31.5	109.1

[1] BW = body weight; n = neutered.

Table S2. Primers used in the qPCR assay.

Target species	Primer	Sequence (5'->3')	Annealing temperature (°C)	Reference
Firmicutes (483 bp)	Firm350f	GGCAGCAGTRGGGAATCTTC	65	[24]
	Firm814r	ACACYTAGYACTCATCGTTT		
Bacteroidetes (414 bp)	CFB555f	CCGGAWTYATTGGGTTTAAAGGG	61	[24]
	CFB968r	GGTAAGGTTCCTCGCGTA		
Clostridium cluster I (231 bp)	CI-F1	TACCHRAGGAGGAAGCCA	61	[25]
	CI-R2	GTTCTTCCTAATCTCTACGCAT		
Lactobacillus spp. (341 bp)	LacF	AGCAGTAGGGAATCTTCCA	61	[26]
	LacR	CACCGCTACACATGGAG		
Bifidobacterium spp. (243 bp)	BifF	TCGCGTCYGGTGTGAAAG	66	[27]
	BifR	CCACATCCAGCRTCCAC		
Enterococcus spp. (144 bp)	EnteroF	CCCTTATTGTTAGTTGCCATCATT	61	[27]
	EnteroR	ACTCGTTGTACTTCCCATTGT		
